# Differences in body mass index trajectories of adolescent psychiatric inpatients by sex, age, diagnosis and medication: an exploratory longitudinal, mixed effects analysis

**DOI:** 10.1111/camh.12575

**Published:** 2022-07-07

**Authors:** Justine Anthony, William Johnson, Anthony Papathomas, Kieran Breen, Florence‐Emilie Kinnafick

**Affiliations:** ^1^ National Centre for Sport and Exercise Medicine, School of Sport, Exercise and Health Sciences Loughborough University Loughborough UK; ^2^ St Andrew's Healthcare Northampton UK; ^3^ Research Centre St Andrew's Healthcare Northampton UK

**Keywords:** Adolescents, psychiatric inpatient, body mass index, longitudinal, growth trajectories

## Abstract

**Background:**

Adolescents in secure psychiatric care typically report high obesity rates. However, longitudinal research exploring the rate and extent of change is sparse. This study aimed to analyse sex differences in longitudinal body mass index (BMI) change for adolescents receiving treatment in a secure psychiatric hospital.

**Methods:**

The sample comprised 670 adolescents in secure psychiatric care. BMI trajectories from admission to 50 months of hospitalisation were produced using sex‐stratified multilevel models. Systematic difference in mean BMI trajectories according to age at admission (14, 15, 16, or 17 years), medication (Olanzapine or Sodium Valproate), and primary diagnosis (Psychotic, non‐Psychotic or Functional/behavioural disorders) were investigated.

**Results:**

Together, males and females experienced a mean BMI increase of 2.22 m/kg^2^ over the 50‐month period. For females, BMI increased from 25.69 m/kg^2^ to 30.31 m/kg^2^, and for males, reduced from 25.01 m/kg^2^ to 23.95 m/kg^2^. From 30 to 50 months, a plateau was observed for females and a reduction in BMI observed for males. Psychotic disorders in males (β 3.87; CI 1.1–6.7) were associated with the greatest rate of BMI change. For medication, Olanzapine in females was associated with the greatest rate of change (β1.78; CI −.89–4.47).

**Conclusions:**

This is the first longitudinal study exploring longitudinal BMI change for adolescent inpatients. Results highlight that individual differences in adolescent inpatients result in differing levels of risk to weight gain in secure care. Specifically, males with psychotic disorders and females taking Olanzapine present the greatest risk of weight gain. This has implications for the prioritisation of interventions for those most at risk of weight gain.

**Key Practitioner Message**

- Adolescents in secure settings experience high obesity rates. This contributes to increased prevalence of physical comorbidities and reduced life expectancy in adulthood.
- Little is known regarding the rate of weight gain and factors contributing to excess weight gain in this population.
- This is the first study to model longitudinal BMI change for adolescents in secure care.
- This study identified that individual factors (sex, diagnosis, medication) may contribute to varying BMI trajectories.
- Provides early indication that patients with psychosis may benefit from early intervention to prevent excessive weight gain.
- As weight gain is greatest in the first 12 months, physical health interventions should be offered from admission.

## Introduction

Adolescents with severe mental illness (SMI), experience high obesity rates (Johnson, Day, & Moholkar, 2018). Research suggests 84% of those receiving inpatient treatment gain weight during the first 6 months (Carney, Bradshaw, & Yung, 2018), with 80% becoming overweight or obese (Haw & Bailey, 2012). This weight gain presents significant risk for the development of noncommunicable physical comorbidities such as diabetes, metabolic syndrome and cardiovascular disease (Llewellyn, Simmonds, Owen, & Woolacott, 2016) which contribute to a reduced life expectancy of 15–20 years in those with SMI (Walker et al., 2015).

Pharmacological treatments may increase obesity risk in adolescents (Barker et al., 2018). These are the first line of treatment in inpatient settings to stabilise symptoms which are likely to be at their most severe at admission. Adolescents with SMI are susceptible to their negative metabolic impacts, as a result of being medication naïve; individuals with no prior antipsychotic use gaining more weight (Bak, Drukker, Cortenraad, Vandenberk, & Guloksuz, 2021) as do individuals who start antipsychotic treatment at a younger age (Manu et al., 2015). Additionally, antipsychotic treated patients experience fatigue and sedation leading to reduced physical activity and increased sedentary behaviour (Vancampfort et al., 2016). Two medications of interest in adolescents are Sodium Valproate and Olanzapine. Sodium Valproate is a drug used to treat epilepsy and bipolar disorder and is known to cause weight gain in children and adolescents through increasing insulin and glucose levels (Kanemura, Sano, Maeda, Sugita, & Aihara, 2012). Olanzapine is a second‐generation antipsychotic, known for weight gain in children and adolescents (Fleischhaker et al., 2008) with side effects including reduced satiety and sedation (Ratzoni et al., 2002).

Risk of weight gain from antipsychotics may be secondary to environmental influences. Significant weight gain has been identified in the adolescent inpatient population where only 18% were treated with antipsychotics (Carney, Imran, Law, Folstad, & Parker, 2019). Inpatient environments are described as obesogenic (Johnson, Day, & Moholkar, 2017). Restrictions on movement due to security and safety requirements mean patients spend a significant amount of their day sedentary (Faulkner, Gorczynski, & Cohn, 2009). Additionally, the secure environment may facilitate unhealthy lifestyle choices with suggestions that secure settings may inadvertently promote overeating (Long, Brillon, Schell, & Webster, 2009) with calorie dense and nutritionally poor food being readily available. Additionally, burden of disease is associated with emotional eating where individuals will regularly overeat (Tuncer & Çetinkaya Duman, 2020).

Illness characteristics may present varying levels of risk of weight gain for inpatient adolescents. For example, negative symptoms are associated with weight gain (Cerimele & Katon, 2013). Depression, which has a high prevalence of negative symptoms is associated with obesity, with depressed adolescents 70% more likely to become obese (Mannan, Mamun, Doi, & Clavarino, 2016). Biological factors may contribute, as leptin, an appetite regulating hormone may be dysregulated in depressed individuals (Milaneschi, Simmons, van Rossum, & Penninx, 2019).

Male and females with psychosis also experience rapid weight gain (Teasdale et al., 2018). In psychosis, cognitive deficits, such as impairments in executive functioning have been linked to weight gain, with speculation that impairments in the dopaminergic reward system may lead to overeating (Arnsten & Li, 2005). However, whilst any SMI diagnosis is associated with an increased risk of weight gain, research has not yet established the comparative risk of obesity between disorders.

Regarding sex differences, in both secure and outpatient settings, evidence suggests adult females experience higher obesity rates than males (Coodin, 2001). In adolescent populations, results are mixed. Psychosis has been associated with a greater obesity risk in adolescent males (Cordes et al., 2017) and depression with a greater risk in females (Richardson et al., 2003). There may also be sex differences relating to medication use. For example, Olanzapine, a commonly prescribed antipsychotic is more likely to cause obesity in females than in males (Kraal, Ward, & Ellingrod, 2017). However, reasons underlying these sex differences remains unknown.

Weight gain in secure settings is likely attributable to multiple interacting factors (Johnson et al., 2018). This is concerning as obesity in adolescence not only predicts adult obesity (Simmonds, Llewellyn, Owen, & Woolacott, 2016), but also the development of metabolic abnormalities in adulthood (Freedman, Mei, Srinivasan, Berenson, & Dietz, 2007). Presence of physical health concerns is a contributing factor to delaying discharge in psychiatric inpatients (Fisher et al., 2001). Metabolic risk factors are also evident, with inpatient adolescents reporting elevated serum glucose and cholesterol, in addition to abnormal HDL levels (Cohen, Bonnot, Bodeau, Consoli, & Laurent, 2012; Grover et al., 2015) highlighting the increased vulnerability of this population to physical health concerns (Carney et al., 2021). Consequently, monitoring and awareness of obesity risks for adolescents in secure settings remains important. Whilst NICE guidance for physical health monitoring exists, research indicates this is only partly adhered to (Carney et al., 2018; Gnanavel & Hussain, 2018) with physical health being insufficiently monitored, particularly in those taking antipsychotic medication (Pasha, Saeed, & Drewek, 2015).

Previous research exploring weight gain in secure settings has used cross‐sectional or pre–post design. These research designs are limited as they only capture a snapshot of physical health parameters. Minimal longitudinal research means that we do not understand the risk that individuals present at admission to excessive weight gain and how weight changes during inpatient treatment. Longitudinal research is necessary to help understand the dynamics of weight change and how outcomes may be affected by individual differences. In these formative years, knowledge of factors associated with the greatest propensity to weight gain may allow for prioritisation of interventions for individuals most at risk of excessive weight gain. This would have the potential to reduce metabolic risk and the subsequent development of metabolic abnormalities in adulthood.

The present study will address this gap in the literature by using mixed‐effect growth models to model longitudinal body mass index (BMI) trajectories for adolescents who are, or have, resided in a large UK psychiatric hospital between 1986 and 2020. Specifically, we will look at the influence that age at admission sex, medication, and primary diagnosis have on BMI trajectories. The aims of this study were to identify:

- Are there differences in BMI trajectories between male and female adolescents in a secure psychiatric setting?
- Are there differences in BMI growth trajectories based on primary diagnosis?
- How does medication use impact BMI growth trajectories?

## Methods

### Setting

The research was conducted in a CAMHS UK psychiatric hospital consisting of medium and low secure wards. At the time of data extraction, there were a total of 8 wards, each with 8 beds within the CAMHS service. Patients within this setting present with a range of diagnoses at a severity that cannot be safely managed through community or outpatient services. Developmental disorders are common with separate wards for learning disorders. The setting does not have a specialised eating disorder unit, but some have eating disorders as secondary diagnoses. Treatment is delivered by a multidisciplinary team involving psychiatrists, psychologists, occupational therapists, speech and language therapy, dietician, physiotherapists and a sports and exercise therapist. Education is provided onsite where patients take part in lessons and sit examinations.

### Data characteristics

We used a secondary retrospective analysis of patients' electronic medical notes. Ethical approval for the analysis of anonymised data was provided by the Human Participants Sub‐committee at an East Midlands University in the UK. The initial dataset prior to data cleaning included 1055 participants who had been admitted to CAMHS between 1986 and 2020. Each patient was assigned an anonymised ID.

The outcome measure was BMI. Within the dataset, this was recorded at irregular intervals by a clinician within the secure setting. Not all participants had their BMI recorded at admission and number of months before the first measurement varied. As each BMI measurement was accompanied with a date stamp, months from admission for each measurement were calculated. If patients had multiple BMI measurements within a month the average of these measurements were calculated, providing a single measure for that month. Eighty‐six participants were excluded that had less than three BMI measurements as participants with only one or two measurements would have provided insufficient information for the model. Although Stata can handle missing data, it was considered a pragmatic decision to exclude participants with less than three measurements as those participants would provide insufficient information to produce a reliable trajectory with. A sensitivity analysis comparing estimates from observations included across the entire sample to those with less than three BMI measurements is within the Supporting Information Table (S4). Hospitalisation time ranged from 0 to 143 months. Initial observation of the data revealed that the majority (90%) of the 7727 BMI observations were in months 0–50 after admission. Therefore, observations outside of this admission range were excluded.

The dataset included participants aged 10–21 years at admission. Initial exploration highlighted that most participants were aged between 14 and 17. Patients outside of this range (118) were excluded as they provided very little data and would not be considered representative of that age group. Diagnoses were initially grouped into categories based on the International Classification of Diseases (ICD‐10) classification. For example, a diagnosis of major depression was classed as a mood disorder. This resulted in eight disorder categories. To reduce the number of parameters in the models, this was reduced to three categories. These included psychotic disorders, nonpsychotic mental disorders, and functional/behavioural disorders (Appendix S1). For example, schizophrenia was classed as a psychotic disorder, major depression as a nonpsychotic disorder and autism as a functional/behavioural disorder.

Data was provided on whether patients were taking either Olanzapine or Sodium Valproate at any point during their treatment. Therefore, this was coded as 0 and 1 within the dataset. Data was excluded if patients had been treated with medication for less than a month. Information was provided on medication dose. However, as administration method varied (e.g., oral vs. injectable) and the fact that dose is dependent on body weight, meant it was not possible to standardise dosage information.

### Statistical analysis

Statistical analysis was conducted in Stata (Stata Press, 2019). Descriptive analyses for demographic information were produced. Mixed effects growth modelling using the mixed command were used to produce BMI trajectories for adolescents from admission to 50 months. Mixed effects growth modelling is an analytical method well suited for longitudinal data (Johnson, Balakrishna, & Griffiths, 2013). It can account for irregular measurement intervals alongside accounting for missing data using a missing at random assumption, which reduces the risk that the parameter estimates are biased.

Mixed effects growth modelling produces a single average curve for the entire sample from the fixed effects, but the addition of random effects also captures the individual deviations from the fixed effects and together the fixed and random effects identify individual trajectories. As longitudinal growth is being measured, the hierarchical structure of the data is such that measurement occasions are nested within individuals. In all the models, measurement occasion (in months following admission) was the level 1 variable. This was set at zero (representing the time from admission to 1 month) and increased in 1‐month intervals. In the model, the intercept represents the average BMI at month 0, whereas the slope represents the extent to which BMI changes each month.

Three separate models were fitted, one for the entire sample, one for males and one for females. The model included a linear and quadratic term for the time variable which were also included as random effects. The inclusion of a quadratic term in the model was justified using the Bayesian Information Criterion, with lower value indicating a better model fit (Wang & Bodner, 2007).

Fixed effects incorporated in the model included age at admission, diagnosis, and medication (sodium valproate, olanzapine, or neither). Interaction variables for each fixed effect were produced with the time variable. These allowed for linear BMI change to differ according to age, diagnosis, and medication. All the fixed effects were included as dummy variables in the model. Age at admission was included as a dummy variable within the model. For age, those admitted at age 14 acted as the reference and those at ages 15, 16, and 17 were included as fixed effects. For diagnosis, functional/behavioural disorders acted as the reference with psychotic disorders and nonpsychotic disorders included in the model. Maximum likelihood estimation was used as the default estimation method and an unstructured covariance matrix was used. A histogram ensured that Level 2 random effects in each model were normally distributed. A formal description of the model is provided in the online Supporting Information (Appendix S2) and the do‐file is available on Figshare (Anthony, 2022). Model selection is presented in Table S5.

In addition to the mixed effects growth model, we reported period effects of the variables in the model. To explore this, we presented the number average BMI based on decade, alongside number of patients diagnosed with disorders in each category, and number of patients treated with either Olanzapine or Sodium Valproate. This is presented in the online Supporting Information (Table S1).

## Results

### Sample characteristics

Average age at admission was 15.8 ± 1.09. Average length of stay was 20.9 ± 12.2 months. Fourteen percent of the sample were taking Olanzapine and 9.8% of the sample were taking Sodium Valproate. Of those with Psychotic Disorders, 10% were taking Olanzapine and 2% were taking Sodium Valproate. Of those with nonpsychotic disorders 14% were taking Olanzapine and 8% were taking Sodium Valproate. Of those with Functional/Behavioural Disorders 15% were taking Olanzapine and 18% were taking Sodium Valproate. The mean BMI across the entire cohort was 28.56 kg/m^2^ ± 6.74, median = 27.5 (IQR = 23.3–32.2) with patients reporting an average BMI increase of 2.22 kg/m^2^ across the 50‐month period. Demographic information for the sample is shown in Table S2.

### Growth model

**Figure 1:**
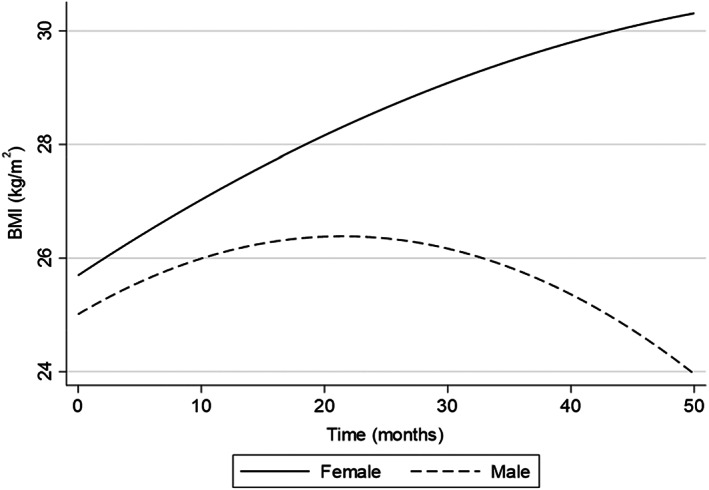
Graph showing body mass index trajectories for males and females

According to the multilevel models, the mean BMI at admission (0 months) was 25.69 kg/m^2^ for females and 25.01 kg/m^2^ for males. BMI rate of change was slightly higher for females (β 0.14; CI −.009–−.28) than males (β 0.12; CI −0.01–2.48). For males, a negative interaction with month (β −.002; CI −.005–−.0007) indicated that the BMI rate of change slightly reduced over time. A small negative interaction with month was also observed for females (β −.001; CI −.003–.001). Figure 1 shows the predicted trajectories for males and females from 0 to 60 months. Table S3 in the online Supporting Information shows multilevel model estimates of BMI trajectories for males and females.

### Medication

At baseline, males taking Olanzapine had a BMI of 25.56 kg/m^2^ and those taking Sodium Valproate had a BMI of 25.53 kg/m^2^. The monthly rate of change was slightly lower for Sodium Valproate (β 0.53; CI −5.03–6.08) than Olanzapine (β 0.55; CI −2.30–3.40). The medication by month interaction was negative for both Olanzapine (β −.07; CI −.18–.03) and sodium valproate (β −.10; CI −.31–.01) indicating that the positive associated of medication with BMI attenuated with age.

**Figure 2:**
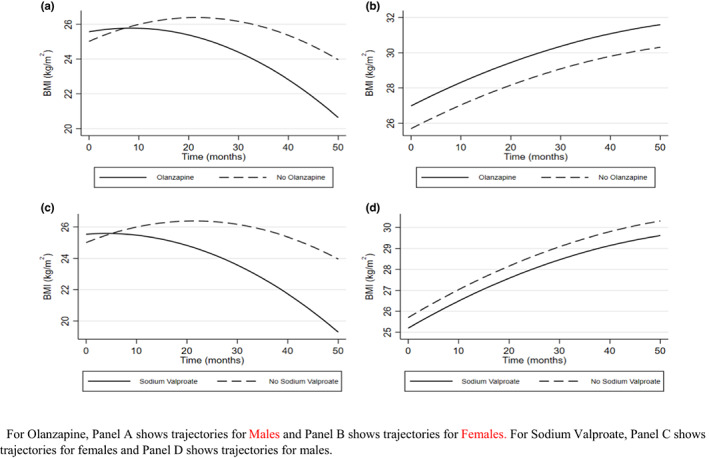
Graph showing body mass index trajectories for patients taking olanzapine and sodium valproate. For Olanzapine, Panel A shows trajectories for Males and Panel B shows trajectories for Females. For Sodium Valproate, Panel C shows trajectories for females and Panel D shows trajectories for males

At baseline, females taking Olanzapine were estimated to have a BMI of 27.47 kg/m^2^ which was associated with a monthly rate of change (β 1.78; CI −.89–4.47). A positive month by medication interaction (β .05; CI 0.07–.169) indicated that rate of change accelerated over time. Females taking Sodium Valproate had an estimated BMI at admission of 25.18 kg/m^2^. This was associated with a negative coefficient (β −.50; CI −3.65–2.65) indicating that rate of change reduced over time. The negative month by medication interaction (β −.003; CI −.14–.13) indicated that the negative association of medication with BMI attenuated over time. Figure 2 shows growth trajectories for males and females taking Olanzapine and Sodium Valproate.

### Diagnosis

For females with nonpsychotic disorders, baseline BMI was estimated at 26.72 kg/m^2^ which was associated with a greater rate of month‐on‐month change (β 1.03; CI −1.23–3.32) than those with functional/behavioural disorders. The rate of change decelerated over time, indicated by the negative interaction with month (β −.03; CI −.13–.07). Similarly, in males, those with nonpsychotic disorders had a higher BMI at baseline 26.47 kg/m^2^, that also increased at a higher rate (β 1.12; CI −1.11–3.36) which, demonstrated by the interaction coefficient, decelerated over time (β −.011; CI −.10–.87).

**Figure 3:**
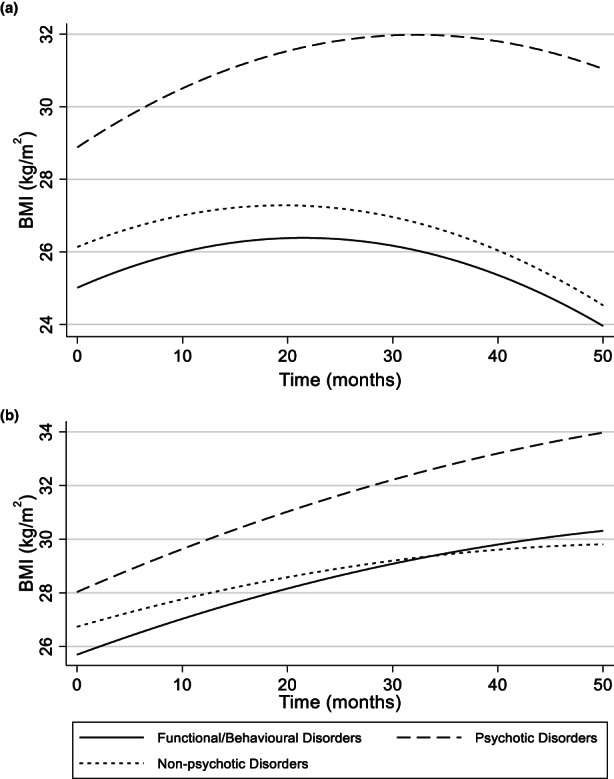
BMI trajectories based on disorder. Panel A shows trajectories for males and Panel B shoes trajectories for females

For females with psychotic disorders, BMI at baseline was estimated at 28.02 kg/m^2^ and associated with a monthly change of (β 2.33 kg/m^2^; CI −.44–5.12), of which the rate of change decelerated over time (β −.03; CI −.13–.07). For males with psychotic disorders, estimated BMI at baseline was 26.43 kg/m^2^ with a large expected monthly change (β 3.87; CI 1.06–6.67) which also accelerated over time (β .06; CI .053–.18). Figure 3 shows the predicted growth trajectories for each disorder category.

## Discussion

This is the first longitudinal study to explore BMI trajectories for adolescents in a secure psychiatric setting. Both males and females reported a BMI increase whilst in secure care. However, for both the rate of change attenuated over time. Females taking Olanzapine and males with psychotic disorders demonstrated the greatest rate of BMI increase throughout the 50‐month observation period.

A significant proportion of the weight gained was during the first 6 months, similar to rates reported in another adolescent inpatient sample (Carney et al., 2019). It is also important to consider how rate of change compares to that in the general population. According to the World Health Organisation BMI reference (Rodd et al., 2014) between the ages of 14 and –17, males report an approximate BMI change of 0.05 kg/m^2^ each month, whilst females report an estimated change of 0.04 kg/m^2^ each month. Comparatively, in the present study, the estimated monthly BMI change was 0.14 kg/m^2^ for females, and 0.12 kg/m^2^ for males, indicating that the rate of change exceeds that reported in the general population. There are several explanations for this. For example, pharmacological treatment is associated with rapid weight gain in adolescent inpatients (Barker et al., 2018), typically prescribed in higher doses at admission. Additionally, during initial months of treatment, symptoms are most severe, meaning capacity to reduce sedentary time and negative health behaviours is diminished (Farholm & Sørensen, 2016).

After the initial increase in BMI, the model predicted that after 30 months the rate of increase plateaued and then appeared to decrease. Specifically, the model predicted that males experience a reduction in BMI between 20 and 30 months following admission, whereas females only experience a plateau during the same time period. The observed decrease in BMI was of interest as it suggests that beyond 30 months, males in this setting may experience a reduction in BMI. The confidence intervals for both males and females monthly change were also relatively small, indicating that we can have some certainty of the preciseness of this effect.

A plateau, or a reduction in BMI should be considered in the context of potential external factors. Over time as the severity of patient's conditions lessens, they may be treated with lower doses of medications. Similarly, as severity of condition improves, then patients may be afforded more freedoms such as leave (Rogers, Papathomas, & Kinnafick, 2019) which may provide opportunities to reduce sedentary behaviour and increase physical activity. Further research is required to determine how sex differences in growth trajectories could potentially relate to diet, medication, and physical activity engagement.

We aimed to understand how individual characteristics influenced growth trajectories. The models predicted males with psychotic disorders would experience greater weight gain relative to nonpsychotic disorders or behavioural disorders. Albeit to a lesser extent, females with psychotic disorders showed a similar pattern. Psychotic disorders in adolescence present numerous risks for weight gain. First, excessive energy intake in adolescent psychosis is common (Teasdale et al., 2018), potentially explained by the prescription of antipsychotics which are associated with reduced satiety and subsequent weight gain (Bak et al., 2021). Cognitive deficits in psychosis may also contribute to weight gain (Arnsten & Li, 2005). However, as only two medications were incorporated in the analysis, disentangling the effects of medication from behavioural risk is not possible.

Males with psychotic disorders appeared to gain weight at a significantly faster rate than females. Research exploring sex differences in metabolic parameters in psychosis has mixed results. Males with schizophrenia may be more likely to engage in health behaviours which increase obesity risk (Goldstein et al., 2007) whilst also experiencing greater deficits in executive functioning (Andreano & Cahill, 2009) which has previously been linked to energy intake (Teasdale et al., 2018). However, other research has identified that whilst males with schizophrenia demonstrate greater risk on metabolic parameters, females report a higher BMI (Kraal et al., 2017). Whilst highlighting the need for further research into sex differences in BMI, this suggests sex could be an independent risk factor for weight gained in secure care that should be considered at admission.

Medication data included Olanzapine and Sodium Valproate. Both have previously been associated with weight gain in adolescent populations (Fleischhaker et al., 2008; Kanemura et al., 2012), at rates similar to those reported in the present study. For medication data, relatively wide confidence intervals (perhaps due to small numbers of patients) indicate that there may be some uncertainty surrounding the estimates of the effect. These may also indicate significantly variability surrounding the effect of medication, a finding previously reported in adolescent inpatients (Fleischhaker et al., 2008). Additionally, uncertainty surrounding comparators i.e., other medications that patients would be receiving mean that we are unable to ascertain the relative and comparative effect of Olanzapine to other medication types. However, considering Olanzapine data alone, females did report more weight gain than males. This finding aligns with research suggesting that the odds of developing metabolic adverse effects may be greater in females (Kraal et al., 2017). The potential risk that adolescent females face indicates that there may be value in exploring alternative medications that have a lower metabolic impact.

Several variables were not included in the analysis could have the potential to influence adolescent growth trajectories. Research has indicated that pubertal status may be relevant, with those who start puberty earlier having a higher BMI in adolescence (Arim, Shapka, Dahinten, & Willms, 2007). Though such information was not available, inability to include these, and other variables may have influenced the perceived impact of effects included within the model. Additionally, it could be considered a limitation that only Olanzapine and Sodium Valproate were the only forms of medication that there was available data for. There are many forms of pharmacological treatment which adolescents may receive whilst in secure care. Future research that can incorporate these may allow us to understand how medications or groups of medications play a role in BMI growth trajectories and allow us to consider the relative impact of these.

There may be some concerns with the generalisability of the data. As the data was collected over a large timespan, it is likely that factors implicating weight gain may have changed over time. Changes in policy, medication and therapy may have changed over time. For example, a call for physical health to be prioritised for those with mental illness was released in 2015 (NHS England, 2014). However, a review of progress made indicated that changes are likely to be gradual (Ham, Buckley, & Baylis, 2016). Context specific factors may have also had an impact on weight gain. For example, lifestyle interventions that may have been implemented on a particular ward. Therefore, it remains a limitation that the factors influencing weight gain may have changed during the observation period and therefore may not be able to be generalised to future cohorts.

Additionally, within the dataset, we could not control for medication history. We recognise that prior to admission within the current setting, patients may have been treated with other medications. This may have implicated findings as research has indicated that adolescents who are medication naïve are more likely to gain weight that those who have had previous exposure to medication (Correll et al., 2009). However, it is also important to recognise that with first exposure to medications with a high propensity to weight gain, NHS guidance indicates that individuals should be frequently monitored and placed on less ‘risky’ medications if metabolic impacts become apparent (NHS England, 2018). Therefore, due to the impact that prior medication utilisation may have, and our inability to control for it within this study, we also recognise this as a limitation of the current work.

There are research and practice implications of our findings. The findings of this study may allow identification those most susceptible to weight gain and thus be prioritised for early intervention. This presents a necessary step in terms of prioritising physical healthcare in those most at risk of adverse health outcomes. By developing an understanding of individual risks, we align with the requirement to provide patient centred care for psychiatric inpatients which aspires to improve inpatient care by focusing on individual needs (Gabrielsson, Sävenstedt, & Zingmark, 2015).

## Conclusions

This is the first study to explore longitudinal changes in BMI for adolescents in a secure psychiatric setting. It has allowed us to foster an initial understanding of the factors and characteristics associated with weight gain, therefore identifying those at greater risk of metabolic adverse effects whilst in secure care. Males with psychotic disorders and females taking Olanzapine were highlighted as highest risk for excessive weight gain for adolescents in secure psychiatric care. These groups may benefit from early intervention in terms of lifestyle interventions and/or medication review to minimise this potential weight gain.

## Ethical information

Ethical approval for the present study was granted by Loughborough University Human Participants Sub‐committee (Date: 12/06/2020 ID: 2020‐1624‐1379).

## Supporting information


**Table S1** Table illustrating period effects for the data.
**Table S2.** Demographic Information.
**Table S3.** Multilevel model estimates of BMI trajectories for males and females.
**Table S4.** Comparison of model parameter estimates of those included in the final sample, versus those with less than three BMI measurements.
**Table S5.** Model Selection.
**Appendix S1.** Disorder Categorisation.
**Appendix S2.** Formal description of mixed effects growth model.
