# Supplementary material for: Differences in body mass index trajectories of adolescent psychiatric inpatients by sex, age, diagnosis and medication: an exploratory longitudinal, mixed effects analysis
Source: Child Adolesc Ment Health. 2022 Jul 7;28(2):318–26. doi: 10.1111/camh.12575 (PMC10946920; doi:10.1111/camh.12575)
Supplement: Supplementary file 1 — Table S1 Table illustrating period effects for the data. Table S2. Demographic Information. Table S3. Multilevel model estimates of BMI trajectories for males and females. Table S4. Comparison of model parameter estimates of those included in the final sample, versus those with less than three BMI measurements. Table S5. Model Selection. Appendix S1. Disorder Categorisation. Appendix S2. Formal description of mixed effects growth model. [file CAMH-28-318-s001.docx]

**Supporting Information**

**Table S1.** Table illustrating period effects for the data.

|  | Decade (n) | | | |
| --- | --- | --- | --- | --- |
|  | 1980 (1) | 1990 (19) | 2000 (164) | 2010 (486) |
| BMI m(sd) | 35.21(1.47) | 27.43(4.50) | 30.01(7.39) | 28.16(6.66) |
| Psychotic (%) | 0 | 2 | 40 | 73 |
| Non-psychotic (%) | 0 | 14 | 91 | 301 |
| Functional (%) | 1 | 3 | 33 | 60 |
| Olanzapine | 1 | 9 | 13 | 45 |
| Sodium Valproate | 1 | 2 | 8 | 19 |

| Male (%) | 53.8 |
| --- | --- |
| Age at admission (years), Mean (SD) | 15.95 (1.09) |
| Length of stay (months), Mean (SD) | 24.8 (18.7) |
| **Ethnicity** | n(%) |
| Asian/Asian British | 12(1.79%) |
| Black/African/Caribbean | 40(5.97%) |
| Mixed/multiple ethnic | 5 (0.75%) |
| White | 497(74.18%) |
| Any other ethnic group | 7(1.04%) |
| Any other mixed, multiple ethnic group | 14 (2.09%) |
| Any other white | 9 (1.34%) |
| Not stated, not known | 86 (12.84%) |
| **Diagnosis at baseline** | % |
| Psychotic mental health disorder | 16.20% |
| Non-psychotic mental health disorder | 69.0% |
| Functional/Behavioural Disorder | 14.30% |
| **Medication** |  |
| Olanzapine | 74 (11.3%) |
| Sodium Valproate | 35 (5.2%) |

**Table S2.** Demographic Information

**Table S3.** Multilevel model estimates of BMI trajectories for males and females. Associations are shown with time, age at admission, diagnosis, and medication*.*

|  | Males |  | Females |  | Difference |
| --- | --- | --- | --- | --- | --- |
| -2Log-Liklihood | 6034 |  | 6760 |  |  |
| Df(n) | 3281(3297) |  | 3831(3847) |  |  |
|  | β (95% CI) | p | β (95% CI) | p |  |
| constant | 25.0(21.9-28.1) | <.005 | 25.69 (22.85-28.54) | <.005 |  |
| Month (since admission) | .12(-.002-0.26) | 0.06 | .14(-.0009-.288) | 0.030 |  |
| Month squared | -.002(0.001- -0.007) | 0.008 | -.001 (-.003-.001) | 0.235 |  |
| Age15 | 1.63(-1.40-4.73) | 0.385 | -.95 (-2.56-2.36) | 0.694 | 0.81 |
| Age16 | 0.46(-2.48-3.41) | 0.646 | -1.13 (-3.71-1.44) | 0.673 | 1.2 |
| Age17 | 1.15(-1.76-4.06) | 0.509 | .04 (-2.32-2.42) | 0.63 | 0.76 |
| Psychotic Disorders | 3.87(1.01-6.67) | 0.005 | 2.33 (-.44-5.12) | 0.108 | 2.73 |
| Non-psychotic disorders | 1.12(-1.11-3.36) | 0.26 | 1.03(-1.23-3.32) | 0.559 | 0.44 |
| Olanzapine | .55(-2.30-3.40) | 0.79 | 1.78(-.89-4.47) | 0.312 | 0.53 |
| Sodium Valproate | .53(-5.03-6.08) | 0.86 | -.50 (-3.66-2.65) | 0.982 | 0.52 |
| **Interaction Effects** |  |  |  |  |  |
| Age15*month | (-.13-1.12) | 0.900 | .07(-.04-.18) | 0.451 | 0.034 |
| Age16*month | .07 (.04-.19) | 0.241 | .001(-.22-.22 | 0.707 | 0.087 |
| Age17*month | .04(-.068-.16) | 0.47 | -.04 (-.15-.06) | 0.340 | 0.092 |
| Psychotic Disorders*month | .06(.053-.18) | 0.239 | .02 (-.10-.15) | 0.749 | 0.048 |
| Non-psychotic disorders*month | -.01(-.10-.08) | 0.934 | -.03(-.13-.07) | 0.657 | 0.02 |
| Olanzapine*month | -.077 (-.12-.03) | 0.243 | -.03(-.07-.17) | 0.375 | 0.116 |
| Sodium Valproate*month | -.10(-.31-.09) | 0.315 | -.003 (-.14-.13) | 0.978 | 0.139 |
| **Random Part** |  |  |  |  |  |
| SD(month) | .42(.37-.47) |  | 0.51 (.45-.56) |  |  |
| SD(month2) | .01(.01-.02) |  | .016(.014-.019) |  |  |
| SD(_cons) | 7.10 (6.50-7.76) |  | 6.21 (5.70-6.75) |  |  |
| corr(month, month2) | -.85 (-.89--.78) |  | -.89(-.92---.84) |  |  |
| Corr(month,_cons) | -.28(-.41--.15) |  | -.38 (-.49--.26) |  |  |
| Corr(month2,_cons) | -.21 (-.41--.15) |  | .26 (.11-.39) |  |  |
| SD(Residual) | 1.01 (.98-1.04) |  | .96 |  |  |

**Table S4.** Comparison of model parameter estimates of those included in the final sample, versus those with less than three BMI measurements.

|  | Included in final sample (n=670) | | Excluded for <3 BMI measurements (n=86) | |
| --- | --- | --- | --- | --- |
|  | Female | Male | Female | Male |
| Month | .14 | .12 | .1451679 | .1253388 |
| Estimated Monthly BMI Change | -.001 | -.002 | -.002 | .002 |
| Age15 | -.95 | 1.63 | .43 | 1.38 |
| Age16 | -1.13 | 0.46 | -.59 | .93 |
| Age17 | .04 | 1.15 | .086 | 1.28 |
| Psychotic Disorders | 2.33 | 3.87 | 1.7 | 2.38 |
| Non-psychotic Disorders | 1.03 | 1.12 | -.26 | -1.22 |
| Olanzapine | 1.78 | .55 | 1.44 | -.241 |
| Sodium Valproate | -.50 | .53 | -.05 | .74 |
| Constant | 25.7 | 25.0 | 26.1 | 26.2 |

**Table S5.** Model Selection

|  | **Males** | | **Females** | |
| --- | --- | --- | --- | --- |
| **Model** | **AIC** | **BIC** | **AIC** | **BIC** |
| Linear Model | 14306 | 14324 | 16624 | 16643 |
| Random Intercept Model | 14172 | 14196 | 16334 | 16359 |
| Model with random intercept & random slope | 12779 | 12816 | 14473 | 14510. |
| Quadratic Model | 12115 | 12269 | 13571 | 13727 |

**Appendix S1.** Disorder Categorisation

**FUNCTIONAL/BEHAVIOURAL**

- Neurodevelopmental Disorders *(atypical autism, childhood autism, aspergers)*
- Asperger’s
- Brain Damage/Chromosomal Disorders *(Anoxic brain damage, foetal alcohol syndrome, Chimera)*
- Conduct disorders *(hyperkinetic conduct disorder, unsocialised conduct disorder)*
- Other developmental disorders (*Mild/moderate mental retardation, cognitive disorder)*
- Social functioning disorders *(attachment disorders, adjustment disorders)*

**PSYCHOTIC DISORDERS**

- Schizophrenic Disorders (*Schizophrenia, schizoaffective disorders, schizoid disorder, schizotypal disorder, catatonic schizophrenia, schizoid personality disorder, paranoid schizophrenia)*
- Psychotic Disorders (*Transient psychotic disorders, schizophrenia-like psychotic disorders, persistent delusional disorders, unspecified non-organic psychosis)*

**NON-PSYCHOTIC DISORDERS**

- Personality Disorders (*Dissocial personality disorder, emotionally unstable personality disorder, anxious avoidant personality disorder, histrionic personality disorder, Paranoid personality disorder)*
- Depressive Disorders (*Recurrent depressive disorder, severe depressive episode, dysthymia, major depressive disorder)*
- Anxiety Disorders (*Specific isolation phobias, social anxiety disorder, organic anxiety disorder, anxiety disorder, obsessive compulsive disorder)*
- Bipolar Disorders *(hypomanic, manic, with depression)*
- Post-Traumatic Stress Disorder

**Appendix S2.** Formal description of mixed effects growth model

$y_{\mathrm{ij}}= \beta_{0ij}+\beta_{1j}x_{\mathrm{ij}} +\beta_{2j}x_{\mathrm{ij}}^{2}$ + $\beta_{3}\left( D_{age15j} \right)+ \beta_{4}\left( D_{age16j} \right)+ \beta_{5}\left( D_{age17j} \right)+ \beta_{6}\left( D_{psychoticj} \right)+ \beta_{7}\left( D_{non-psychoticj} \right)+ \beta_{8}\left( D_{olanzapinej} \right)+ \beta_{9}\left( D_{SVj} \right)+ \beta_{10}\left( D_{age15} \right)\left( x_{ij} \right)+ \beta_{11}\left( D_{age16} \right)\left( x_{ij} \right)+ \beta_{12}\left( D_{age17} \right)\left( x_{ij} \right)+ \beta_{13}\left( D_{psychotic} \right)\left( x_{ij} \right)+ \beta_{14}\left( D_{non-psychotic} \right)\left( x_{ij} \right)+ \beta_{15}\left( D_{Olanzapine} \right)\left( x_{ij} \right)+ \beta_{16}\left( D_{SV} \right)\left( x_{ij} \right)$

$\beta_{0ij}=\beta_{0}+\mu_{0j}+e_{\mathrm{ij}}$

$$\beta_{1j}=\beta_{1}+\mu_{1j}$$

$$\beta_{2j}=\beta_{2}+\mu_{2j}$$

$$\left[ \begin{matrix} \mu_{0j} \\ \mu_{1j} \\ \mu_{2j} \end{matrix} \right] \sim N\left( 0,Ω_{\mu} \right): Ω_{\mu}= \left[ \begin{matrix} \sigma_{\mu0}^{2} & & \\ \sigma_{\mu01} & \sigma_{\mu1}^{2} & \\ \sigma_{\mu02} & \sigma_{\mu12} & \sigma_{\mu2}^{2} \end{matrix} \right]$$

$$\left[ e_{\mathrm{ij}} \right] \sim N\left( 0,Ω_{e} \right): Ω_{e}= \left[ \sigma_{e}^{2} \right]$$

$y_{\mathrm{ij}}$ is the BMI at month $i$of patient $j$. $\beta_{0ij}$ and $\beta_{1j}x_{\mathrm{ij}}$ have both fixed and random effects ( $\mu_{0j}$ and $\mu_{1j}$). $\beta_{3}-\beta_{16}$ are the regression coefficients. $D$ denotes the regression coefficient as a dummy variable. For example, $D_{olanzapinej}$ is a dummy variable coded as 1 if a patient was receiving Olanzapine, and 0 if they were not.

$\sigma_{\mu0}^{2}$, $\sigma_{\mu1}^{2}$ and $\sigma_{\mu2}^{2}$ represent the variances of the random effects, with $\sigma_{\mu01}$, $\sigma_{\mu12}$ and $\sigma_{\mu02}$ being the covariances between the random effects.
